# Semantic Annotation of Predictive Modelling Experiments

**DOI:** 10.1007/978-3-030-61527-7_9

**Published:** 2020-09-19

**Authors:** Ilin Tolovski, Sašo Džeroski, Panče Panov

**Affiliations:** 8 University of Bari Aldo Moro, Bari, Italy; 9 Aristotle University of Thessaloniki, Thessaloniki, Greece; 10 Open University of Cyprus, Nicosia, Cyprus; 11 Dalhousie University, Halifax, NS Canada; 12 Hasso Plattner Institute, Potsdam, Germany; 13 Department of Knowledge Technologies, Jožef Stefan Institute, Ljubljana, Slovenia; 14 Jožef Stefan International Postgraduate School, Ljubljana, Slovenia

**Keywords:** Computational experiments, Semantic annotation, Ontology, Predictive modelling

## Abstract

In this paper, we address the task of representation, semantic annotation, storage, and querying of predictive modelling experiments. We introduce OntoExp, an OntoDM module which gives a more granular representation of a predictive modeling experiment and enables annotation of the experiment’s provenance, algorithm implementations, parameter settings and output metrics. This module is incorporated in SemanticHub, an online system that allows execution, annotation, storage and querying of predictive modeling experiments. The system offers two different user scenarios. The users can either define their own experiment and execute it, or they can browse the repository of completed experimental workflows across different predictive modelling tasks. Here, we showcase the capabilities of the system with executing multi-target regression experiment on a water quality prediction dataset using the Clus software. The system and created repositories are evaluated based on the FAIR data stewardship guidelines. The evaluation shows that OntoExp and SemanticHub provide the infrastructure needed for semantic annotation, execution, storage, and querying of the experiments.

## Introduction

Data mining and machine learning experiments are conducted in higher volume than ever before, in various settings and domains. In the case of predictive modelling, the users usually aim to produce a model that will provide the best predictive performance. However, in practice, almost none of the settings regarding the experimental setup are stored. We usually do not keep track of the exact software environment, the exact dataset that was used to train the model, the duration of the experiment, and the hardware specification of the machine the experiments were performed on.

The same problem arises when it comes to the models produced by the performed experiments. Regarding the algorithm setup, almost no information is stored about the parameter values of the algorithm implementation which produced the models, and the evaluation scenario used to validate the results. These predicaments make the conducted research hard to verify, reproduce and reuse. There have been previous efforts to address this problem such as the ones developed by Vanschoren et al. [18], Google (AI hub)^1^, Schelter et al. [14], and others.

Having access to a repository of computational experiments that are represented by a schemata based on logical formalism is beneficial from several perspectives. First, the results can be accessed, easily verified, and predictive models can be retrieved for their further reuse. We can utilize the logic behind the schema to pose queries that will allow searching not only through the explicit axioms that are asserted but also on the implicit axioms that the reasoners have produced. From this, we can derive new information based on results already stored in the knowledge base.

However, producing the experiments and then transforming their outputs into this logical formalism can be a tedious and error-prone task when repeated for each experiment. Therefore, one can assume that having a framework that will execute the experiments and format the output according to the defined logical formalism, i.e., ontology-based annotation schema, store the annotations in a database, which will be open for querying through a query endpoint, will provide an easy access to a vast knowledge base of experimental workflows, benefiting both data mining practitioners and domain experts. In the literature, there had been efforts for development of ontological resources that allow semantic representation of different entities in the domain of data mining and machine learning. Examples of state-of-the-art resources OntoDM [12], DMOP [9], Exposé [17], MEX [7], MLSchema [6] and others.

The paper is organized as follows. In Sect. 2, we introduce an ontology module for semantic representation of predictive modelling experiments named OntoExp. Next, in Sect. 4, we demonstrate the use of OntoExp within SemanticHub, a system for execution, semantic annotation, storage and querying of experiments. In Sect. 5, we showcase the use of SemanticHub in a water quality prediction use case scenario. Finally, in Sect. 6, we evaluate the system and the created repository according to the DANS FAIR questionnaire, and in Sect. 7 we give our concluding remarks.

## Representation of Experiments with OntoExp

To create a repository of semantically annotated predictive modeling experiments, we need to create a more granular representation of a predictive modeling experiment that will enable annotation of the experiment’s provenance, algorithm implementations, and results.

For this purpose, we introduce OntoExp, an extension of OntoDM-core [12] for representation of predictive modeling experiments. OntoExp provides a representation of different types of predictive modeling experiments on the execution level. Each experiment type, as well as all of the involved entities and processes, need to be formally represented and connected to provide an annotation schema that will used to produce a comprehensive metadata for the experiment.

The main focus of OntoExp is on representing different types of experimental data mining workflow executions, including the executions of different algorithm implementations together with their parameter setup for various data mining tasks. A connection is made with the inputs and outputs of the execution process, i.e., the datasets, predictive models, and experimental results as concretizations of the evaluation measure implementations. In Table 1, we outline the competency questions that are addressed by our developed extension.

**Table 1.** Examples of competency questions addressed by OntoExp.

#	Competency question
1	List all experiments that address the multi-target regression task
2	List all experiments that used an ensemble learning algorithm
3	List all experiments that had a specific dataset, given as input
4	Return the best predictive model learned on a specific given dataset
5	List all experiments that used cross-validation

**Ontology Design.** OntoDM [12] was developed in a modular fashion, making it suitable for extension. It adheres to the Open Biomedical Ontologies (OBO) Foundry principles [16] for ontology design. These include the use of an upper-level ontology, formal ontology relations, absence of orphan classes, single inheritance, as well as integration and reusing of terms that are already defined in other ontologies. It is based on the Basic Formal Ontology (BFO) [1] as the upper-level ontology, and the relations are reused from the Relations Ontology (RO) [15]. Furthermore, OntoDM reuses classes defined in other ontologies which are relevant to the domain, such as the Information Artefact Ontology (IAO) [3], OntoDT ontology of datatypes [13], Software Ontology (SWO) [11], Ontology of Biomedical Investigations (OBI) [2], and others. All of the reused classes from these ontologies are imported following the Minimum Information to Reference an External Ontology Term (MIREOT) [5] principles.

OntoExp builds on top of the current ontology structure following the same class taxonomy, as well as design and class reuse principles. Supporting the modularity of OntoDM, it is designed as a separate module that can be used by itself or preferably together with OntoDM for a more comprehensive representation of the domain. The ontology module consists of 296 classes in total, 146 of which are novel classes, and 150 are reused from OntoDM. Since the resource is not introduced as a novel standalone ontology, it is licensed under the same license as OntoDM. The ontology module is available at the following PURL https://w3id.org/ontoexp.

**Core Classes.** OntoExp follows the Algorithm-Implementation-Execution Design Pattern and principles defined by Lawrynowicz et al. [10]. The most important classes are *algorithm*, *algorithm implementation*, and *algorithm execution*.

Data mining algorithms are represented as a subclass of the general *algorithm* class from the IAO ontology [3] and represents a specification of an algorithm. We distinguish between DM algorithms that output a single model, and the ones that output an ensemble of models.

*Algorithm implementation* is a concretization of a algorithm specification, implemented in some software product, and written in a specific programming language. In the ontology, we also explicitly represent the provenance information for both the software and the programming language.

An *algorithm execution* is a process that represents the training part of a predictive modeling experiment. It realizes the *algorithm implementation*, receives a *DM-dataset* with a *train set role* as an input, and outputs a *predictive model*. This process precedes the *predictive model execution* that represents the process which realizes the *predictive model* in order to output the *DM-dataset* with the predicted values of the target variables.

**Fig. 1.**
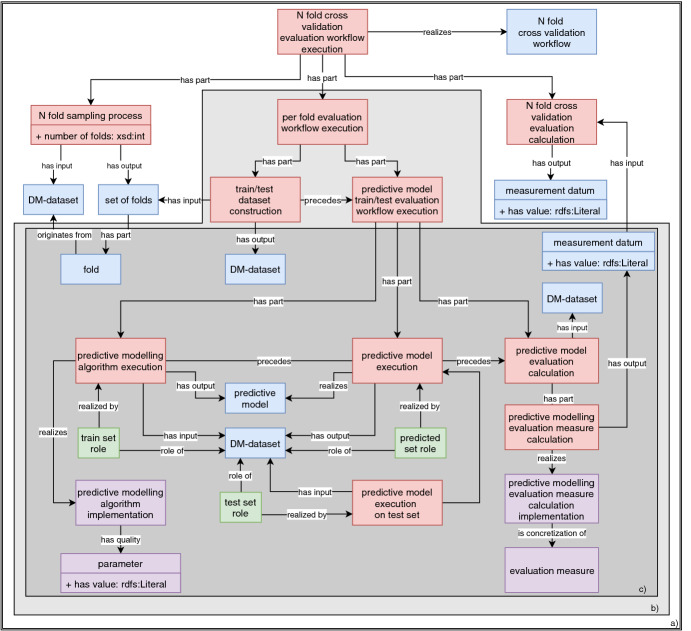
A representation of a predictive modeling experiment: a) N-fold cross-validation scenario. b) Representation of the evaluation for each fold. c) Representation of the train/test evaluation workflow execution. Red boxes represent processes, blue boxes represent information entities, green boxes represent roles and pink boxes represent realizable entities. (Color figure online)

Following the *predictive model execution* (see Fig. 1c), there is the *evaluation calculation* process which uses the predicted *DM-dataset* from the *predictive model execution* process as an input to calculate a specific implementation of an *evaluation measure*. Depending on the type of experiment, or task, the calculation process can vary in different ways. If the experiment has a N-fold cross-validation model evaluation, we need to represent each per-fold *evaluation measure calculation*, and calculate the average value across all measurements. Additionally, if we have complex tasks, such as for example multi-target prediction task, we need to calculate the evaluation measures for each target separately. More details and examples are provides further on in Sect. 3.

**Workflow Representation.** In OntoExp, we represent the *predictive model evaluation train/test workflow execution* and the *N-fold cross-validation workflow execution* processes and their inputs and outputs. The first workflow can use either a separate test set for evaluation or validation of results on the training set, while the second uses N-fold cross-validation as the evaluation method.

*N-fold cross-validation workflow execution* contains the *predictive model train/test evaluation workflow execution* as one of the sub-process. In a cross-validation scenario (see Fig. 1a), we first perform the sampling of the dataset on N folds, and in each iteration (see Fig. 1b), we build a predictive model on N−1 folds and evaluate it on the one fold that was not used for training. Finally, at the end we calculate the average average value of the evaluation measure from all the folds. These repetitive evaluations are represented by the *per fold evaluation workflow execution* process (see Fig. 1b) which consists of two sub-processes, i.e., *train/test dataset construction*, and *predictive model evaluation workflow execution*. Each per fold evaluation process is a sub-process of the *N-fold cross-validation workflow execution* process connected to it with the has part relation.

**Fig. 2.**
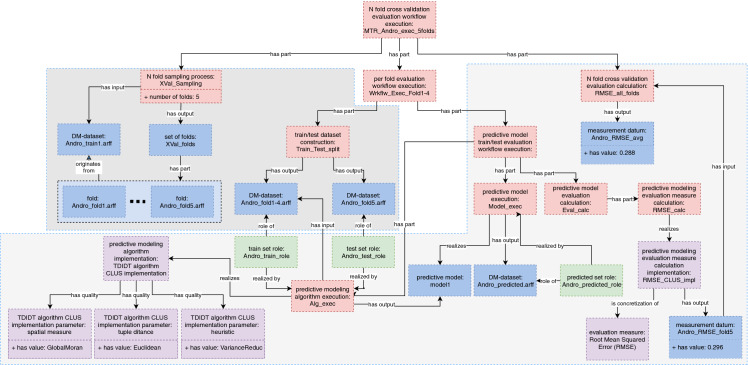
An example of semantically annotated multi-target regression experiment.

## Semantic Annotation of Experiments Using OntoExp

In this section, we describe the complete annotation schema derived from OntoDM and OntoExp on an example of an experiment that involves a cross-validation evaluation for a multi-target regression task using an algorithm that solves that task. In order to represent a cross-validation experimental scenario, we use the *N-fold cross-validation evaluation workflow execution* class (see Fig. 2). This evaluation scenario consists of three consecutive processes: data sampling process, model construction process, and model evaluation process.

First, we focus on the data sampling process represented with the *N-fold sampling process*. We relate this process with a datatype property that carries information about the number of folds the data should be split in. The input of the process is the original *dataset* used for the experiment, while the output is a *set of folds* which is related to each *fold* that will be used for the model evaluation process.

Another part of the data sampling process are the different combinations of folds used for the training and testing purpose. We represent this with the *train/test dataset construction*, which is a part of the *per-fold evaluation workflow execution* and outputs two *DM-datasets*, one that consists of N−1 folds and will be used with a *train set role*, and the one fold to be used with a *test set role*.

Next, in a cross-validation scenario, there is a separate model creation and evaluation process for each fold. This is then repeated N times, N being the number of folds. To represent this, we use the *per fold evaluation workflow execution* class. This process consists of two parts, *train/test dataset construction*, which we introduced before, and *predictive model train/test evaluation workflow execution*. The latter is the process that connects the model creation and model evaluation process for a given training and test set. The resulting output of the model creation process is a predictive model.

Next is the evaluation process, which starts with a *predictive model execution* process that uses the already built predictive model to produce a *dataset* with the predictions for the target variables. This dataset is then used to calculate the evaluation metrics for each target, since we are dealing with the task of multi-target prediction. The evaluation measures are always dependent on the task at hand. This is a part of the *predictive model evaluation calculation* process.

Finally, once these calculations are finished for all folds, we use them as an input of the *N-fold cross-validation evaluation calculation*, which then calculates the averages for each target across all folds, and also the final average value across the final per-target values.

## System for Executing and Querying Predictive Modeling Experiments

In this section, we present SemanticHub, a web-based system for remote experiment execution, semantic annotation, storage, and querying of predictive modeling experiments. The presented system provides an infrastructure for running experiments on a remote server, annotating their outputs and experimental settings, storing the raw files in a file storage system, and the annotations in a triple store database. The stored annotations are available for querying either through a user interface or using a querying endpoint. The prototype version of the system is available at http://semantichub.ijs.si/clus/experiment.

**Fig. 3.**
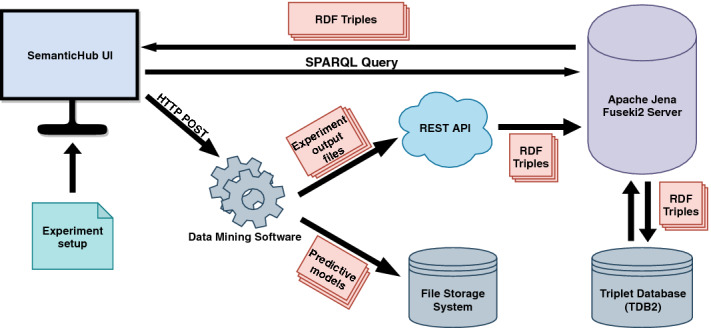
The architecture of SemanticHub.

**System Architecture.** SemanticHub is constructed in modular fashion as a synthesis of several independent web services (see Fig. 3). First, the input datasets are sent to our file storage through the a FTP server. The experiment is defined through interaction with SemanticHub’s UI, which sends the parameters and the setup to our server where our data mining software is hosted. Currently, we are using the Clus software^2^ for executing the experiments. Clus is a decision tree and rule induction system that implements the predictive clustering framework and has been applied to many different tasks including multi-task learning, structured output learning, multi-label classification, hierarchical classification, and time series prediction.

The whole setup as well as the experimental outputs are annotated with entities and processes defined in OntoExp using a REST API. The annotated experimental setup, metrics, and results are sent to the Fuseki2 server^3^ as sets of RDF triples^4^. The resulting predictive models are stored as raw files the file system. The Fuseki2 server hosts the triple-store database which is used for storage and retrieval of the RDF triples. These triples are available to the users through SemanticHub’s querying engine, which generates SPARQL queries based on the user’s input. The results are shown to the users in SemanticHub’s UI.

**Running Computational Experiments.** Here, we describe the implementation of the framework that allows users to set up and execute computational experiments on our remote servers. One of the two user scenarios for this system allows users to run their own experiments. This is done in two stages. First, the user needs to define the experimental setup, by uploading an experimental specification or setting up the experiment through the user interface. This step includes selecting the datasets, the algorithm for training the models, as well as its parameter values. The dataset is uploaded through a HTTP request to a repository that is open to the users through FTP requests.

For running predictive modeling experiments we use the Clus software for data mining. As examples here, we focus on the tasks of single-target regression and classification, as well as multi-target regression and multi-label classification. For these tasks, the software has two main inputs: a settings file, and the datasets, both for training and testing purposes. The settings file contains the complete experimental setup, i.e., it defines the input data, specifies which part of it is descriptive and which are the target attributes, as well as all of the model constraints and algorithm parameters (for more details please check^5^). Depending on the criteria set in the settings file, Clus can output predictive models, experimental results, as well as predictions for each test example.

The user interface for setting up and executing an experiment in the Clus software consists of a single screen, where the user needs to upload the train and test datasets, as well as the settings file. Additionally, there are two checkboxes, one for the selection of the validation scenario, and one for defining whether a single model or an ensemble algorithm will be run on top of the selected data. Each of these settings/flags runs Clus in a different mode, changing the number of output files, as well as the type of the output files.

**Fig. 4.**
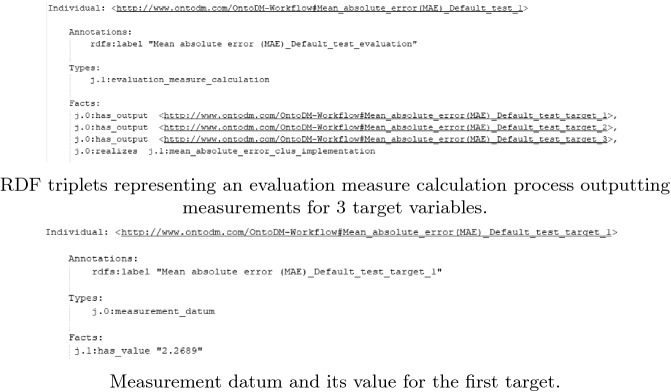
Examples of annotations of experimental results.

**Semantic Annotation Workflow.** Following the experiment execution, the system utilizes the designed annotation schemata for predictive modeling experiments to create semantic annotations in the form of RDF graphs. The RDF graphs consist of triplets representing the inputs and outputs of the experiment, the algorithm used, its parameter values, as well as the evaluation results (see Fig. 4). Formalized in this way, the RDF graphs are then uploaded to a TDB2 triplet database hosted on a Fuseki2 server. The upload is executed by the SPARQL Graph Store HTTP Protocol.

The CLUS library we use for running predictive modeling experiments provides a comprehensive output, once the experiment is completely finished. Thus, we semantically annotate the experiments after the execution of the experiment. The complete settings file with all default, and user-defined values are contained in the output file for each experiment, enabling us to annotate the experimental setup, runtime provenance information, and results in one step. We should also note that the annotations are solely based on the annotation schemata designed for predictive modeling experiments that use the OntoExp ontology.

**Fig. 5.**
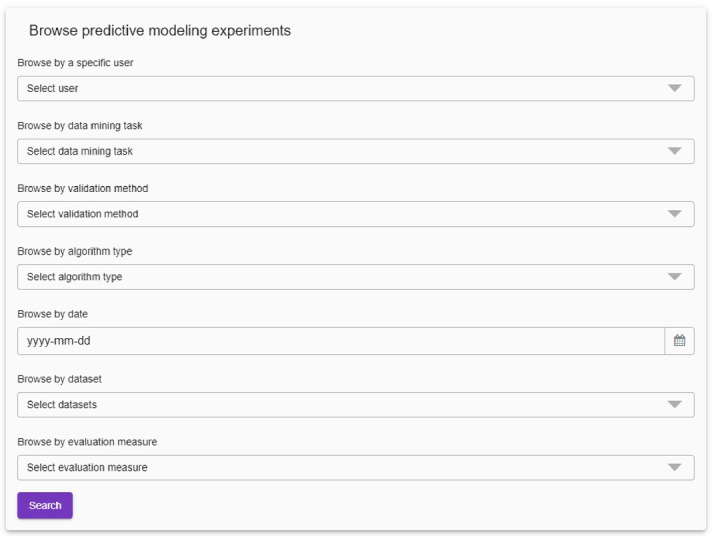
An example of querying interface for CLUS experiments.

**Querying the Repository of Predictive Modeling Experiments.** The second user scenario of our system is the one where users can query or browse through the database of completed experiments. We use the Graph Store HTTP Protocol for storing and querying the semantic annotations, which are in the form of RDF graphs. The SPARQL endpoint provides the presence on the HTTP network for receiving and handling Graph Store HTTP or SPARQL Protocol requests. The SPARQL querying interface enables users to write raw SPARQL queries directly for each RDF dataset. However, our system provides a simple graphical user interface, where users can define their queries by interacting with the user interface.

For the predictive modeling experiments conducted in the Clus framework, users can query the repository of experiments based on several criteria. These include the user, the data mining task that was addressed, the validation method, algorithm type, datasets included, evaluation measure, as well as the date or range of dates when the experiment was conducted. The querying screen from the user interface is shown in Fig. 5. All of the fields allow multiple selections, therefore, the query result can be a set of experiments, not just a single instance.

## Use Case: Water Quality Prediction

In this section, we present a use case scenario of SemanticHub’s predictive modeling system integration with the Clus data mining framework. Specifically, we will showcase the data import, construction of the experimental setup through the settings file, as well as the remote execution of an experiment. Finally, we will use SemanticHub’s SPARQL endpoint access to formulate a SPARQL query.

**Fig. 6.**
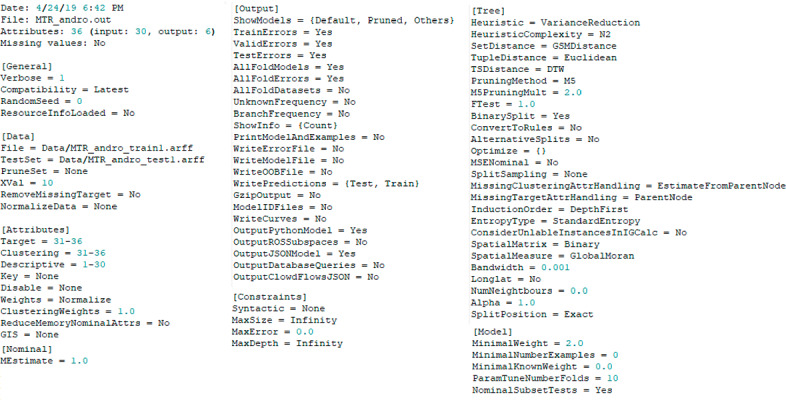
A specification of the experiment (a screenshot of a Clus settings file).

We define the experiment specification through the settings file, as shown in Fig. 6. Here, we provide information about the input datasets for this experiment, together with the parameters and constraints for the model that will be trained. Namely, we will use the Andro datasets^6^ for water quality prediction [8] for training a single multi-target predictive clustering regression tree. Additionally, we define the descriptive, clustering, key, and target variables. The datasets contain 30 descriptive, as well as 6 target features (temperature, turbidity, oxygen, pH, conductivity, salinity). Regarding the model constraints, we do not limit the tree size in terms of depth, or the minimum number of examples in the leaf (see Fig. 6). We choose the variance reduction heuristic for making the splits, with $N^{2}$ complexity. Additionally, we set the rest of the parameters with their default values recommended by the Clus development community.

Finally, we define the output settings, i.e., the verbosity of Clus. Normally, in this section, the user can choose which resources are to be stored for memory optimization. However, in this case, our system overrides the user’s preference and selects the settings for maximum verbosity. Doing so, we can successfully annotate not only the experimental setup but the experimental outputs as well.

Once the users have set their experimental setup, the data and settings files are uploaded through SemanticHub’s API in our file system. At this point, the experiment is set and the execution has begun. The user is notified when the execution has finished with a server response.

**Fig. 7.**
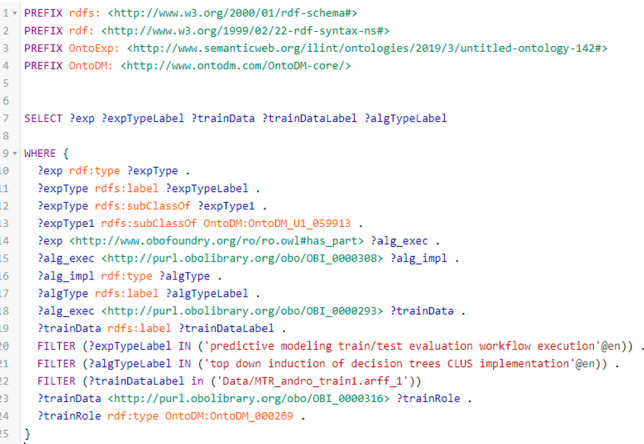
Generated SPARQL query in the SPARQL endpoint for the experiment ran in Clus.

Once a user has completed the experiments, we can use SemanticHub’s SPARQL endpoint to browse through the repository of completed experiments. For example, we can formulate a query that returns details for the experiment executed previously. For this purpose, we search for an experiment that has the Andro dataset as input, addresses the multi-target regression task, has a train-test evaluation scenario, and outputs a single model. The generated SPARQL query, by the UI, is shown in Fig. 7.

## Evaluation According to the FAIR Guidelines

The FAIR principles are focused on the findability, accessibility, interoperability, and reproducibility of the resources [19]. Here, we evaluate our experiment repositories based on the checklist^7^ for evaluation of data FAIRness introduced by the Data Archiving and Networked Services (DANS). We can distinguish between five types of questions, regarding the trustworthiness, findability, accessibility, interoperability, and reproducibility of the repository. In Table 2, we present the assessment questions and discuss the results of the evaluation.

**Table 2.** Evaluation questions from the DANS FAIRness assessment

	Data trustworthiness	Answer	Points
Q1	Is the data repository you have chosen trustworthy?	Yes	2/4
	**Data findability**	Answer	Points
Q2	Will your dataset have a Persistent Identifier after deposit?	Yes	1/1
Q3	Did you provide enough information (metadata) about your data for others to understand and reuse your data?	Yes	1/1
Q4	Did you provide rich additional documentation?	Yes	1/1
	**Data accesibility**	Answer	Points
Q5	Is the metadata publicly accessible?	Yes	1/1
	**Data interoperability**	Answer	Points
Q6	Are the data stored and archived in preferred archival formats?	Yes	1/1
Q7	Did you use standardized vocabulary?	Yes	1/1
	**Data reusability**	Answer	Points
Q8	Did you give detailed provenance information for the data?	Yes	1/1
Q9	Do you make use of relevant community standards?	Yes	1/1
Q10	Does the data have a usage licence?	Yes	1/1

**Trustworthiness Assessment.** Since we strongly abide by the FAIR principles, we cover the questions of public findability and accessibility of our repository of computational experiments. Additionally, we provide metadata that enables reproducibility of the experimental results, together with raw files that allow reusability of the trained models. However, one additional criteria for the trustworthiness is the CoreTrustSeal [4] certificate that unfortunately we have not obtained yet, hence we obtain two out of four points for this assessment (Q1).

**Findability Assessment.** Both our resources and our repository, have persistent URIs (Q2). Next, the annotation schemata introduced in Sect. 2 provides a comprehensive representation of the experiments in the domain of predictive modelling, together with essential provenance information such as creator, date, software environment, hardware capabilities of the machine, etc. (Q3). Finally, we (can) provide additional documentation in the form of a link to the publication where a certain computational algorithm was introduced (Q4).

**Accessibility Assessment.** All of the metadata stored about the conducted computational experiments, as well as the ontologies describing them, are publicly available through SemanticHub’s querying interface, and the SPARQL endpoint hosted on the Fuseki2 server (Q5).

**Interoperability Assessment.** The metadata that we generate for the computational experiments is stored in the RDF format. This format is preferred in the knowledge representation and semantic web community. Additionally, RDF has several syntax variations and the users can switch between different syntax models to their preference (Q6). For semantic annotation, we used ontologies designed by following the state-of-the-art best practices in ontology engineering. All of the resources, are publicly available and uniquely identified (Q7).

**Reusability Assessment.** We provide provenance data for each computational experiment regarding the creator, software environment, hardware capabilities, as well as the date of the creation of the experiment (Q8). Since we create and are in full control of the data that enters our repository, we make sure that the generated metadata is in consistent format, which was previously determined to follow community standards regarding the information it contains (Q9). Finally, all of our resources, as well as the metadata in the experiment repository are published under the Creative Commons CC 4.0 usage license, which enables free use for all non-commercial use provided the work is referenced (Q10).

**Summary.** To evaluate our input for this questionnaire, each of the questions participate with one point in the final score with the exception of the first question regarding the trustworthiness of the repository which has a score of 4 points. For this question, we achieve 2 out of the 4 possible points, since we have not yet obtained the CoreTrustSeal certificate. Regarding the data findability, since we have positive score on all three questions, we achieve 3/3 points. For the accessibility of our repository, we achieve 1/1 point. Storing the data in community-preferred and versatile archival format combined with the use of standardized vocabulary helps us score 2/2 points for the interoperability of our metadata. For the reusability of our metadata for computational experiments we score 3/3 points since we have affirmative answers to the listed questions. In total, we achieve 11/13 points for this assessment.

## Conclusion

In this paper, we focus on the semantic representation and annotation of predictive modelling experiments. First, we outlined the need and the benefits of creating a semantically annotated repository in the domain. We proposed OntoExp, a resource that provides a semantic representation for each conducted experiment. In addition, we incorporate OntoExp in SemanticHub, a system that can execute, annotate and store the experiments. The conducted experiments in the system are annotated and stored in a TDB2 triplet database hosted on Fuseki2 server. SemanticHub allows for these experiments to be executed through its own infrastructure, meaning that the users can define and run the experiments on our physical servers. In addition, we provided a querying interface from which the users can query the repositories of experiments. Finally, we evaluated the produced experiment repository according to the DANS FAIRness checklist.

In future work, we plan to upgrade this prototype system with more functionalities. These will include the use of different software platforms to execute the experiments and building a user management module. We also plan to use the representational power of ontologies and reasoners to enhance the system’s querying engine and capabilities.

## Footnotes

1 https://cloud.google.com/ai-hub/.

2 http://clus.sourceforge.net/doku.php.

3 https://jena.apache.org/documentation/fuseki2/.

4 https://www.w3.org/TR/rdf11-primer/.

5 http://clus.sourceforge.net/doku.php?id=doc:main.

6 https://www.openml.org/d/41392.

7 https://tinyurl.com/yyx5uc5k.

## References

[1] Arp R, Smith B, Spear AD (2015). Building Ontologies with Basic Formal Ontology.

[2] Bandrowski A (2016). The ontology for biomedical investigations. PLOS One.

[3] Ceusters, W.: An information artifact ontology perspective on data collections and associated representational artifacts. In: MIE, pp. 68–72 (2012) 22874154

[4] Coretrustseal for data repositories (2019). https://www.coretrustseal.org

[5] Courtot M (2011). MIREOT: the minimum information to reference an external ontology term. Appl. Ontol..

[6] Esteves, D., Lawrynowicz, A., Panov, P., Soldatova, L., Soru, T., Vanschoren, J.: ML schema core specification. W3C (2016). http://www.w3.org/2016/10/mls

[7] Esteves, D., et al.: MEX vocabulary: a lightweight interchange format for machine learning experiments. In: Proceedings of the 11th International Conference on Semantic Systems, pp. 169–176 (2015)

[8] Hatzikos EV, Tsoumakas G, Tzanis G, Bassiliades N, Vlahavas I (2008). An empirical study on sea water quality prediction. Knowl. Based Syst..

[9] Keet CM (2015). The data mining optimization ontology. J. Web Semant..

[10] Lawrynowicz A, Esteves D, Panov P, Soru T, Dzeroski S, Vanschoren J (2017). An algorithm, implementation and execution ontology design pattern. Adv. Ontol. Des. Patterns.

[11] Malone J (2014). The Software Ontology (SWO): a resource for reproducibility in biomedical data analysis, curation and digital preservation. J. Biomed. Semant..

[12] Panov P, Soldatova L, Džeroski S (2014). Ontology of core data mining entities. Data Min. Knowl. Discov..

[13] Panov P, Soldatova LN, Džeroski S (2016). Generic ontology of datatypes. Inf. Sci..

[14] Schelter, S., Böse, J.H., Kirschnick, J., Klein, T., Seufert, S.: Automatically tracking metadata and provenance of machine learning experiments. In: Machine Learning Systems Workshop at NIPS (2017)

[15] Smith B (2005). Relations in biomedical ontologies. Genome Biol..

[16] Smith B (2007). The OBO Foundry: coordinated evolution of ontologies to support biomedical data integration. Nat. Biotechnol..

[17] Vanschoren, J., Soldatova, L.: Exposé: an ontology for data mining experiments. In: International Workshop on Third Generation Data Mining, SoKD-2010, pp. 31–46 (2010)

[18] Vanschoren J, Van Rijn JN, Bischl B, Torgo L (2014). OpenML: networked science in machine learning. ACM SIGKDD Exp. Newslett..

[19] Wilkinson MD (2016). The fair guiding principles for scientific data management and stewardship. Sci. Data.

